# Owl airfoil aerodynamic noise sources and performance compared to hawk and NACA0012 airfoils for low Reynolds applications

**DOI:** 10.1038/s41598-025-06309-x

**Published:** 2025-07-02

**Authors:** M. Kazemi, M. Mani

**Affiliations:** Aerospace Engineering Department, Amirkabir University of Technology, Tehran, Iran

**Keywords:** Owl airfoil, Wind tunnel testing, Low Reynolds flow, Aero-noise, Hawk airfoil, Aerospace engineering, Mechanical engineering, Fluid dynamics

## Abstract

The investigation of low Reynolds number flows is crucial, particularly for applications such as wind turbines and small-scale UAVs. This study compares the owl airfoil with the NACA0012 and hawk airfoils through wind tunnel testing, utilizing pressure sensors and force balance to examine the aerodynamic noise sources and aerodynamic performance of the airfoils. A total of six airfoils were investigated at various Reynolds numbers from 44 × 10^3^ to 160 × 10^3^, considering the glide flight envelop for various owl species. Wind tunnel test results showed higher Cl and L/D ratio for the owl airfoil, outperforming the NACA0012 and hawk airfoils by up to 6.7% and 44.1%, respectively. This is attributed to the optimal camber of the owl airfoil compared to the two other airfoils, and its lower relative thickness too. This helps flight with this airfoil at lower AOA, which reduces noise. In addition, the stall angle for owl airfoil was ranging from 8° to 15° higher than NACA0012 airfoil, which stalled at 10°-11°, and higher than 6° to 12° hawk stall angle. This feature allowed owls to perform efficient flights in glide phase at lower AOA that minimized the main aerodynamic noise sources such as the separations and pressure fluctuations. Pressure measurements represented the initiation of LSB for the owl airfoil at around AOA = 6° to 10° at different Reynolds numbers, while the hawk airfoil shown the presence of LSB starting from AOA = 0°. A detailed analysis of the pressure fluctuations showed that the owl airfoil had fewer sources of aerodynamic noise, such as LSB, stall phenomena, and separated shear layers, on both its upper and lower surfaces, compared to other types of airfoils. Additionally, an analysis in the frequency domain showed that the amplitude of FFT for NACA0012 and hawk airfoil is generally higher compared with the owl airfoil. These findings shed light on the aerodynamic characteristics and noise generation mechanisms of owl airfoil for future research and design considerations.

## Introduction

Many engineering applications, such as small-scale air-vehicles inspired by birds to compact wind turbines and spoilers on road vehicles, operate within a Reynolds number range of 10 × 10^3^ to 200 × 10^3^. This range falls under the category of low Reynolds number flow regime, where fluid behavior is primarily characterized by smooth, laminar flow patterns dominated by viscous forces^1,2^. In this context, Carmichael^3^ conducted an experimental and numerical investigation that categorized various air vehicles based on their flow Reynolds numbers. According to this study, insects, birds, and model airplanes typically operate at Reynolds numbers below 100 × 10^3^. Additionally, wind turbines, particularly those of the VAWT type, fall into this low Reynolds number category. Lower Reynolds number flows exhibit higher drag coefficients and lower lift coefficients due to several factors, including laminar flow, laminar-to-turbulent transition, laminar separation bubbles, strong adverse pressure gradients, and local flow separation regions. Thus, a comprehensive understanding of flow physics and aerodynamic performance in low Reynolds number flows is crucial^4^.

Numerous studies have explored the complexities of low Reynolds number flows over the years. Among these, Toppings and Yarusevych^5,6^ focused on the impact of wing tip shapes on laminar separation bubbles at a Reynolds number of 125 × 10^3^. Their findings revealed that the behavior of the laminar separation bubble formed at inner sections of the wing model, rather than at the tip, mirrors that of a 2D airfoil. This underscores the importance of specifically studying LSBs in airfoil configurations rather than entire wings. Additionally, Grande et al.^7^ investigated how laminar separation bubbles affect noise generation in a propeller at lower Reynolds numbers. Their results demonstrated that shedding of flow from laminar separation bubbles leads to the production of high-frequency noises. Furthermore, Istvan et al.^8^ examined the influence of free-stream turbulence intensity ranging from 0.1 to 2 percent on LSBs. They concluded that increasing turbulence intensity results in earlier transition and reattachment, as well as a reduction in the length of the LSB. Research on low Reynolds number flows has been diverse and extensive. For instance, Maughmer et al.^9^ recently examined an airfoil at Re = 100 × 10^3^, investigating the impact of artificial turbulators on drag reduction in combination with flap effects. Similarly, Singh et al.^10^ focused on designing a wind turbine airfoil across Reynolds numbers ranging from 38 × 10^3^ to 128 × 10^3^, aiming for enhanced performance. Laitone^11^ contributed to the field by studying various airfoils at Reynolds numbers below 70 × 10^3^. Meanwhile, Alam et al.^12^ explored the wake characteristics of a NACA 0012 airfoil across Reynolds numbers ranging from 5 × 10^3^ to 43.5 × 10^3^ through velocity measurement and flow visualization. They noted that the formation of LSB was not observed until Reynolds numbers reached 10 × 10^3^. Furthermore, Genc et al.^13^ conducted research on the NACA 4412 airfoil, investigating the influence of membrane surfaces on airfoil performance enhancement within wind turbine applications. Their study encompassed Reynolds numbers from 21.5 × 10^3^ to 75 × 10^3^.

As previously mentioned, birds, due to their small size and flight velocity, operate at relatively lower Reynolds numbers. Research on bird flight dates back to Storer’s investigation^14^, highlighting the importance of drawing inspiration from avian flight. Aldheeb et al.^15^ conducted a review paper focusing on the performance of bird airfoils specifically at lower Reynolds numbers, underlining the significance of this subject. This topic has garnered attention in the field of unmanned aerial vehicles (UAVs) as well. Harvey et al.^16^ addressed it in a review paper, emphasizing birds’ higher camber and thin airfoils as points of interest for UAV design. Moreover, other studies have shown that the phenomenon of laminar separation bubbles, extending from the leading edge to a turbulent reattachment flow region, increases in length with increasing angle of attack. If the reattachment point coincides with the trailing edge of these airfoils, it could mark the location of maximum lift generation^17–19^.

Another significant application of low Reynolds number airfoils lies in their use for Mars aircraft. Designing Micro Aerial Vehicles (MAVs) for the Martian atmosphere presents unique challenges, primarily due to its low density, resulting in Reynolds numbers typically ranging from 10^3^ to 10^4^. The reduced density and Reynolds numbers lead to diminished lift forces and airfoil performance, challenges that can be mitigated to some extent by Mars’ lower gravity^20^. Studies by Yang et al. have investigated the limitations of rotorcraft on Mars, highlighting cambered thin airfoils as superior candidates for rotorcraft design^21–23^. Building on this research, Kondo et al.^24^ drew inspiration from owl airfoils and conducted an investigation at Re = 23 × 10^3^. They demonstrated that the owl airfoil, characterized by its circular leading edge, straight upper surface, and substantial lift capabilities, could be well-suited for low Reynolds number applications. Additionally, they observed the presence of a laminar separation bubble (LSB) on this airfoil at this Reynolds number, a phenomenon also noted in Aono et al.’s study^25^. Recently, owl airfoil owl airfoil has been the subject of new researches and applications such as ducted wind turbines^26^, Small scaled wind turbines^27^, Simulation of the flow around airfoil^28^, and Flapping motion studies^29^.

As it is reviewed the comprehensive study of airfoils at low Reynolds numbers holds significant importance for various engineering applications, including wind turbines, mars aircraft, and bin inspired technologies based on avian aerodynamics. While previous research has extensively investigated the owl wing properties, particularly the serrated shape of the leading and trailing edges, there remains a gap in the investigation of the behavior of the airfoil shape. However, while other valuable studies have explored the applications of the owl airfoil, they lack a focused investigation into the fundamental flow physics around the airfoil, particularly in relation to aerodynamic noise sources. Additionally, the behavior of the owl airfoil at different Reynolds numbers, especially in the range of 40 × 10^3^ to 160 × 10^3^, has been overlooked, despite this range covering most low Reynolds number flow applications. Additionally, a comparison between the owl airfoil, known for its silent flight, and the hawk airfoil, known for its noisy flight, is missing in the literature. Thus, an experimental investigation involving three distinct airfoils of owl, hawk, and NACA 0012, with varying chord lengths has been undertaken. The chord-based Reynolds numbers for these six airfoils ranging from 44 × 10^3^ to 160 × 10^3^. The experiments encompass a wide range of angles of attack, spanning from pre-stall to post-stall conditions, to elucidate the flow physics across different regimes. The primary objectives of this project are twofold. Firstly, to find out the aerodynamic characteristics of the owl airfoil, renowned for its silent flight, in comparison to the hawk airfoil, known for its noisy flight. Secondly, to contrast the performance of the bird-inspired airfoils with the NACA 0012 airfoil, chosen for its symmetrical and conventional design, to gain deeper insights into flow behavior.

## Methodology and experimental setup

**Fig. 1:**
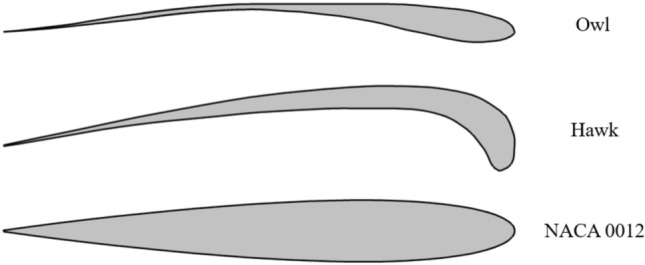
Shape of the three different airfoils used in this study.

As was previously mentioned, three airfoil models (Fig. 1) with two different chord lengths of 15 cm and 25 cm have been chosen for this study, including NACA 0012, Owl airfoil, and Hawk airfoil by the shapes based on previous studies^30,31^. These airfoils were selected to first understand the aerodynamic behavior of owl airfoil and second, to compare its aerodynamic performance with other types of airfoils. The owl airfoil is the main focus of this study due to its distinctive features, including a circular leading edge, a straight upper surface, and substantial lift capabilities, making it well-suited for low Reynolds number applications. The test conditions at which this study was performed are tabulated in Table 1. According to this table, a broad range of angles of attack have been chosen for the purpose of studying attached flow, pre-stall flow, stall, and post-stall flow. Also, different free stream velocities corresponding to Reynolds numbers of 44 × 10^3^ to 160 × 10^3^ are chosen for studies. These velocities and Reynolds numbers are selected to cover the owl glide flight Reynolds number^32^ (Table 2). Additionally, efforts were made to select velocities for the two different chord lengths in such a way that the same Reynolds number was achieved.

**Table 1:** Wind tunnel experiments parameters.

Parameter	Values
Airfoils	Owl, hawk, and NACA 0012
Chord length	15 cm, and 25 cm
Wind tunnel velocities for C15	5.5, 8, 9.2, 10, 12, 13.3, 15, 16.7, and 20 m/s
Wind tunnel velocities for C25	4.8, 5.5, 6, 7.2, 8, 9, 10, and 12 m/s
Reynolds numbers × 10^3^ for C15	44, 64, 74, 80, 96, 107, 120, 134, and 160
Reynolds numbers × 10^3^ for C25	64, 74, 80, 96, 107, 120, 134, and 160
Angles of attack	− 6 to 16°

**Table 2:** Summary of flight data of different owl species^33^.

Bird	Mean velocity (m/s)	Min velocity (m/s)	Max velocity (m/s)
Common kestrel	5.2	3.8	6.2
Harris hawk	5.3	4.2	6.4
Barn owl	5.4	4.6	6.7

**Fig. 2:**
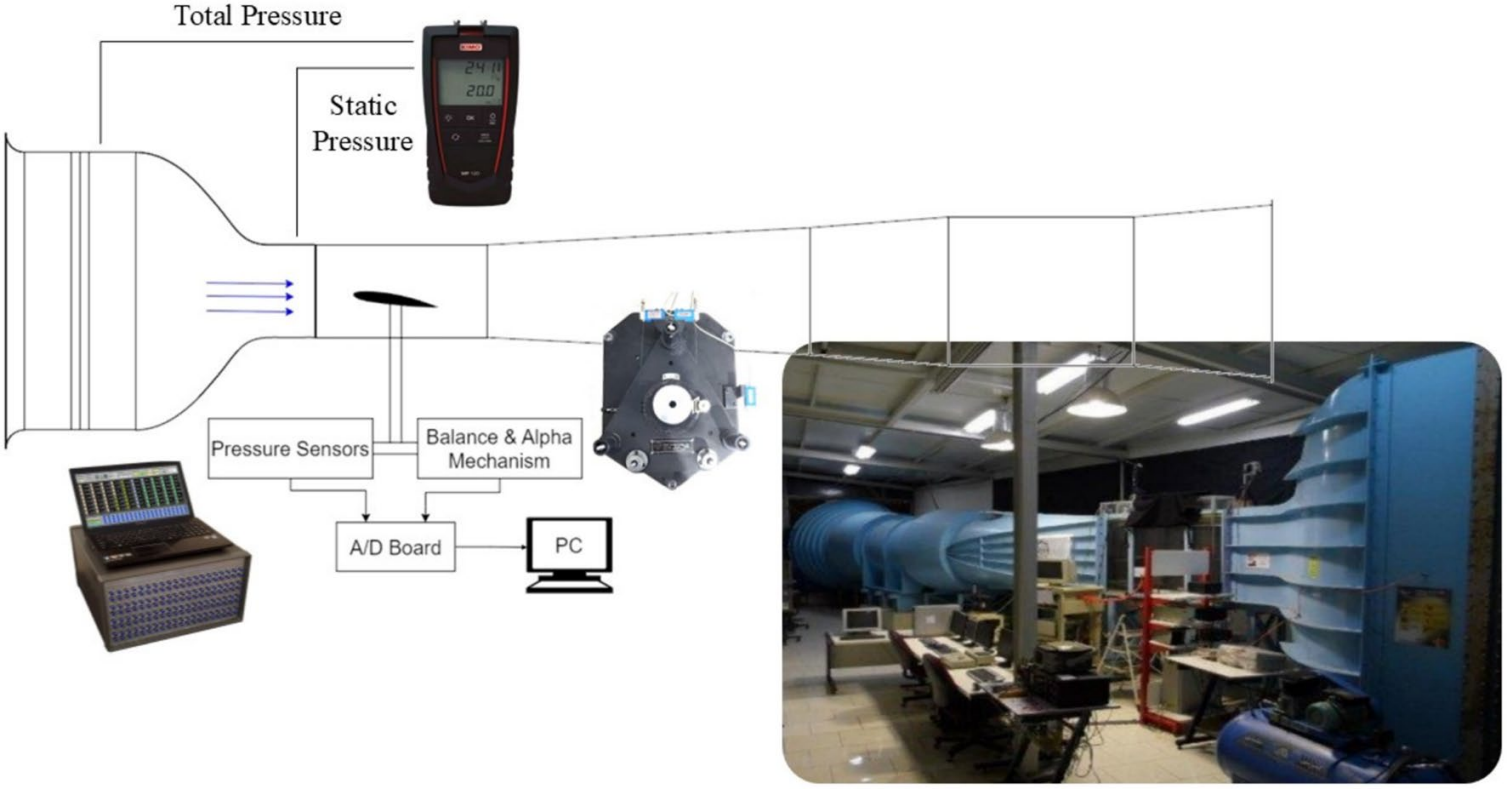
Schematic of the wind tunnel and experimental set-up including the pressure box, force balance, and digital manometer picture.

The measurements were carried out in the low-speed open-circuit wind tunnel with a rectangular test section of 1 × 1 × 1.8 m^3^, contraction ratio of 9:1 and turbulence level as low as 0.1% at DANA Aerodynamic Laboratory at Amirkabir University of Technology. Honeywell pressure sensors (HSCDD025MDNN5) with the maximum range of 5 mbar and full-scale accuracy of 0.1% were used for static pressure measurements. Measurements of the static pressure were carried out at different pressure taps positions distributed over the middle span of the airfoils. To ensure accurate results for pressure distribution, and to eliminate the effect of unfavorable noises, a time window of 30 s with 2 kHz sampling frequency were considered. The sampling frequency is selected based on the facility limitations. Generally, the force load cells have a low frequency response (below 200 Hz) and the extracted data is time-averaged, making frequency less impactful. The Honeywell pressure sensors, however, can measure up to 1 kHz. Data acquisition was performed using a National Instruments PCI6255 A/D card with a 16-bit resolution and a maximum sampling rate of 1.25 MS/s for 30 channels or more than 40 kHz for each channel. As A/D card supports higher sampling rates per channel, but due to sensor limitations, measuring beyond 2 kHz would introduce repeated data. This would grant minimum 576 flow-through times and maximum 4000 flow-through times over the model for the highest and lowest free-stream velocity tested and FFT analysis up to f = 1 kHz based on Nyquist–Shannon sampling theorem. The lift and drag forces applied to different airfoils, were measured using an external three-component balance of TE81 manufactured by Plint and Partners Ltd attached to the vertical wall of the wind tunnel test section. The forces acting on the model are transmitted by cables to three strain gauged load cells with the capacity of 10 kg for the Lift component and 5 kg for the Drag component and a combined full-scale error of less than 3 g. For setting up the velocity in wind tunnel test section, a digital micromanometer namely MP120 from KIMO company is used by measuring the pressure difference between test section and settling chamber and calculating the velocity by accuracy of 0.1 m/s. Load cell calibration by conduction a test and facilities for loading in a standard procedure for receiving the linear relation for each load cell and correction of the blockage effect on the force measurement have been conducted according to Pope and Rae^34^. Figure 2 shows the wind tunnel, data acquisition system and facilities pictures. With respect to the mentioned systematically error values and considering Eqs. (1), (2), ± 0.000181 and ± 0.007531 maximum error bars would be obtained for calculating the force coefficient and pressure coefficient, respectively. Also, based on the repeatability tests a maximum of ± 0.005 and ± 0.01 differences in the value of measured force coefficient and pressure coefficient were observed which was higher than the calculated errors. Thus, it can be concluded that all values of C_F_ and C_P_ in this paper have ± 0.005 and ± 0.01 error bars that are neglected to show because of the better understanding in the presented figures.

1 $${C}_{F}=\frac{\text{F}}{\frac{1}{2}\rho {V}^{2}S}=\frac{\text{F}}{(\frac{1}{1-\frac{{{A}_{TS}}^{2}}{{{A}_{SC}}^{2}}})({P}_{SC}-{P}_{TS})S}$$

2 $${C}_{P}=\frac{\text{p}-{p}_{\infty }}{\frac{1}{2}\rho {V}^{2}}=\frac{\text{p}-{p}_{\infty }}{(\frac{1}{1-\frac{{{A}_{TS}}^{2}}{{{A}_{SC}}^{2}}})({P}_{SC}-{P}_{TS})}$$

## Results

In this chapter, experimental results obtained for three different airfoils of an owl, a hawk, and NACA 0012 at two different chord lengths of 15 cm and 25 cm will be described within the subsequent paragraphs. These experiments were conducted in several Reynolds numbers from 44 × 10^3^ to 160 × 10^3^ and a broad range of angles of attack (− 6° to 16°). These test conditions are motivated by representing flow at lower angles of attack, separation, pre stall and post stall states. Aerodynamic parameters, including Cl, Cd, and L/D of tested airfoils through balance measurement, pressure coefficient distribution, pressure fluctuations contours, and FFT of captured signals will be described thoroughly.

**Fig. 3:**
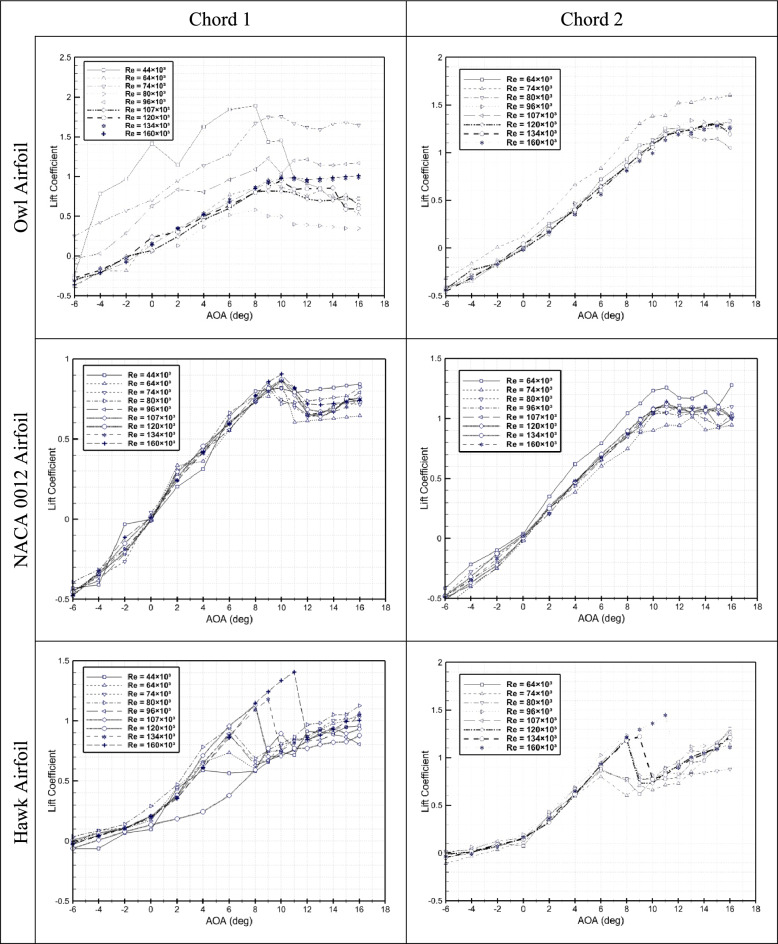
Lift coefficient versus AOA for three airfoils of owl, hawk, and NACA 0012 at two different chord lengths of 15 cm and 25 cm at different Reynolds numbers.

Figure 3 presents the lift coefficient of three airfoils versus angle of attack for two different chord lengths. For the owl airfoil case, the lift coefficient generally decreased by increment of Reynolds numbers especially at the attached area, which is in a direct response to the owl airfoil shape and its high cambered leading-edge. Also, it is visible that the higher lift is for lower Reynolds number of 44 × 10^3^ (V = 5.5 m/s) for model with chord 1 where the behavior is not linear similar to higher Reynolds numbers which could be the effect of laminar boundary layer and free stream low momentum flow on the airfoil. In this figure, a drastic drop in Cl is observed for the angle of attack between 2° and 6° (different by Reynolds number) which could be due to the onset of laminar separation bubble. This behavior is completely eliminated for owl airfoil by increasing Reynolds number. Also, it should be noted that, the non-linear shape of cl diagram for owl airfoil is not visible for Reynolds number higher than 80 × 10^3^ (V = 10 m/s) for chord 1 airfoil. Where the lift of this Reynolds is lower than all Reynolds numbers by a drastically drop. It is very interesting that for both chords of the owl airfoil, the higher lift is for free stream velocity of 5.5 m/s. (Re = 44 × 10^3^ for chord 1 and Re = 74 × 10^3^ for chord 2), which shows that due to high camber of the owl airfoil, the phenomenon at lower velocities, is not follow the Reynolds number as a non-dimensional number. For Reynolds number higher than 80 × 10^3^ in both chords, the flow is predictable and Cl increases with AOA in a linear trend. Also, it is worthwhile to mention that the stall angle for the owl airfoil is delayed by increment of Reynolds numbers from 8° at Re = 44 × 10^3^ to 15° at Re = 160 × 10^3^ due to increment of free stream momentum and separation delay from leading edge. Furthermore, reduction of Cl_α_ and Cl_α=0_ and increment of α_Cl=0_ from − 1.5° to 0.5° for chord 1 from − 2° to 0° for chord 2 at higher Reynolds numbers guarantees more longitudinal stability within these conditions. This happens due to delaying local separations on the surface of airfoil by increment of Reynolds numbers. Based on results presented in Fig. 3, for NACA 0012 airfoil, unlike owl airfoil, Cl is increased by increment of Reynolds number however some cases show different phenomena such as deflection in Cl at the angle of attack of 2° to 6° for Re = 44 × 10^3^, which could be the impact of LSB generation on the upper surface. The results of cl of chord 2 shows that the lowest tested Reynolds number (Re = 64 × 10^3^) have higher lift coefficient due to laminar flow and attachment to all the surface after that for higher Reynolds of Re = 74 × 10^3^ a massive drop happened where could be the effect of boundary layer transition to turbulent. The stall angle for chord 1 is 10° and for chord 2 is 11° which is the direct impact of increasing tests velocity is model with higher chord. Where again prove that just Reynolds number in the range of related to transition could not present the all aspects of the physics of flow. Figure 3 also presents the plots of Cl versus AOA for the Hawk airfoil, considering two different chord lengths. Hawk airfoil Cl trend is very different from two previous airfoils of owl and NACA 0012 due to its high camber and special shape. Interesting thing in this airfoil is stall manner where by increasing the Reynolds number, the stall angle is differed in opposite of two previous airfoils. Stall angle for this airfoil, at both chord 1 and 2 is increased from 6° at Re = 44 × 10^3^ to 11° at Re = 160 × 10^3^. This is the direct impact of the free stream momentum for overlapping to the very high pressure gradient of this airfoil. Generally, the Cl_α_ is the same for different Reynolds numbers but some deflection is visible at AOA 0° to 6° at Re = 44 × 10^3^ and Re = 64 × 10^3^ which could be the effect of LSB generation at upper surface of airfoil. Also, an interesting phenomenon for this airfoil is increasing the lift in post stall area where could be the effect of high camber at lower surface and generating high pressure region in higher angles of attack for this airfoil. In sum, from investigation of lift coefficient plots, it is concluded that the owl airfoil at these range of Reynolds numbers, have higher lift coefficient compared to hawk airfoil and NACA 0012 airfoil. Also, the stall angle of NACA 0012 is better than two others but the owl airfoil is very better than hawk airfoil which could have a direct impact on the noise generation of this airfoil. These results indicate that the relatively moderate camber of the owl airfoil, compared to the hawk airfoil, contributes to better performance in flow physics and outperforms the NACA 0012 symmetrical airfoil.

**Fig. 4:**
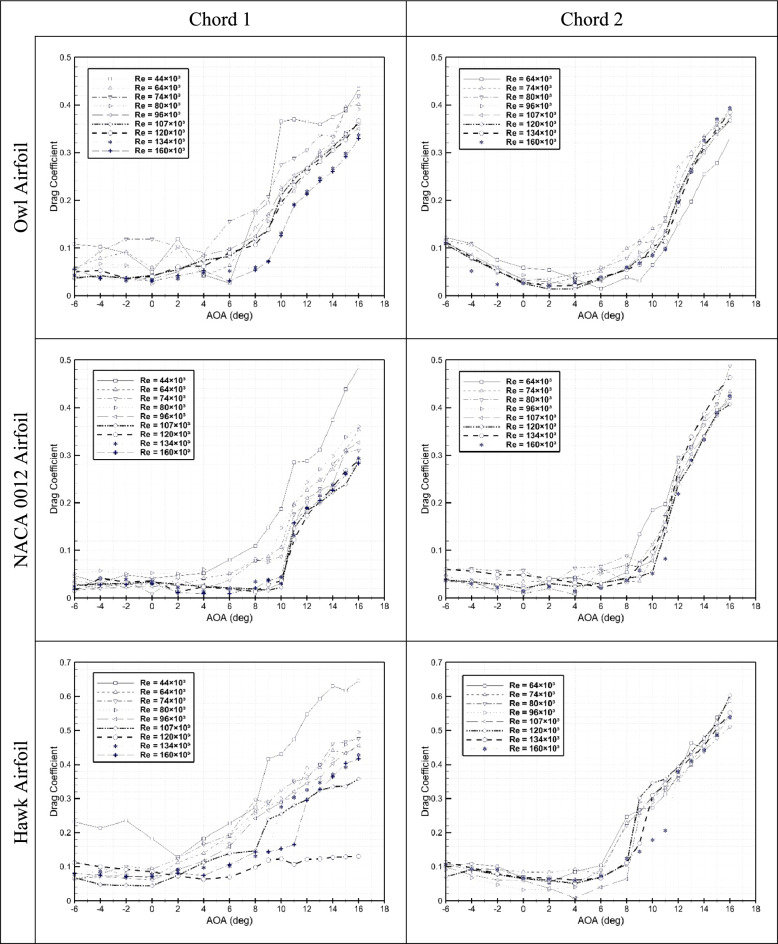
Drag coefficient versus AOA for three airfoils of owl, hawk, and NACA 0012 at two different chord lengths of 15 cm and 25 cm at different Reynolds numbers.

Drag coefficient versus AOA for owl, hawk, and NACA 0012 airfoils with two chord lengths are presented at different Reynolds number from Re = 44 × 10^3^ to Re = 160 × 10^3^ at Fig. 4. Owl airfoil with chord 1 at lowest Reynolds number has a peak at AOA = 2° which could prove the idea of LSB generation on the upper surface of owl airfoil. This phenomenon at this angle of attack is observed for just two lowest tested Reynolds numbers of Re = 44 × 10^3^ and Re = 64 × 10^3^ and is eliminated in higher Reynolds numbers. the highest Cd for angle of attack lower than 8° is for Re = 74 × 10^3^ where it is visible at Cl plots that this Reynolds number could be the situations that the boundary layer is transitioned from laminar to turbulent. Also, after AOA = 8° the lowest Reynolds number of Re = 44 × 10^3^ have highest Cd which is the effect of boundary layer thickening in laminar flow. The Cd plot of chord 2 for owl airfoil shows that the drag coefficient is reduced by increasing Reynolds number for AOA < 4°; however, for higher angles of attack the lowest Reynolds number has lowest drag coefficient which it could be the direct impact of turbulent boundary layer at this Reynolds number by increment of angle of attack. Drag coefficient of NACA0012 airfoil with two different chord lengths at different Reynolds numbers are also presented in Fig. 4. Generally, from the Cd plots it is concluded that for NACA 0012 airfoil, by increment of Reynolds number, the Cd is reduced. Also, for the AOA = 4° an increment is visible at the Cd plot where could be the effect of LSB generation on the upper surface of airfoil which is visited at Cl plot also at these range of angles of attack. Furthermore, in Fig. 4, the plots of Cd for hawk airfoil with two different chord lengths are represented. As it is visible for chord 1 Cd plots, highest drag coefficient is for lowest Reynolds number of Re = 44 × 10^3^, and generally it is concluded that similar to NACA 0012 airfoil and owl airfoil before AOA < 4° is reduced by increment of Reynolds number. Also, a sudden increment in cd is visible for angle of attack higher than 6° which is show the stall phenomena in hawk airfoil where is explained before in Cl plots. However, for Reynolds number higher than 44 × 10^3^, the increment firstly started from 0° which is the effect of LSB generation from 0° in this airfoil.

**Fig. 5:**
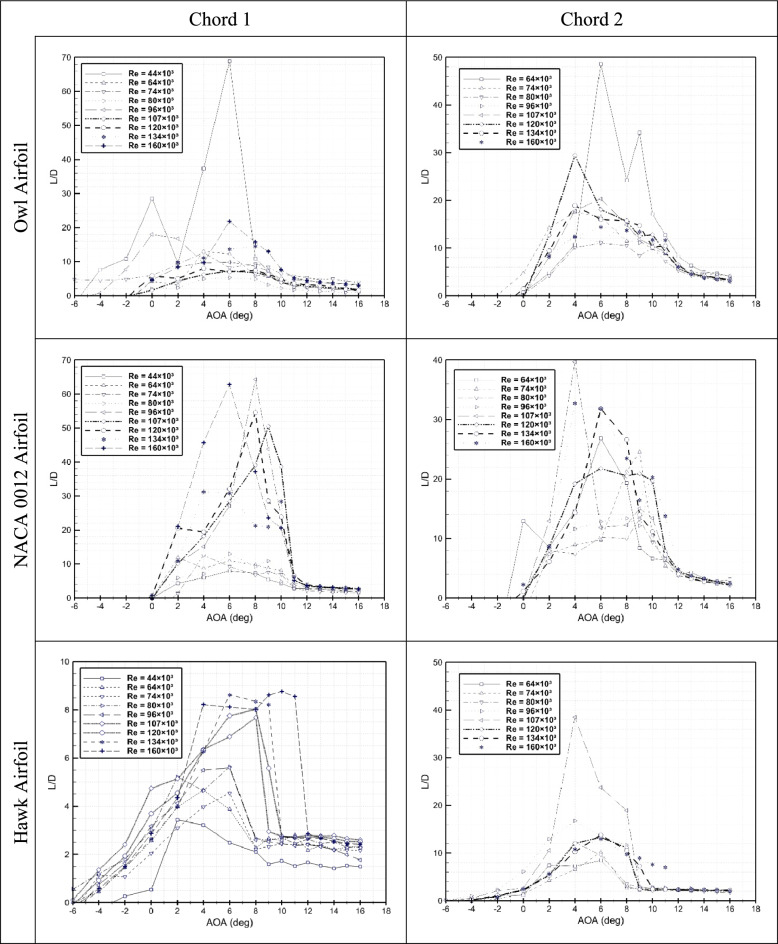
L/D versus AOA for three airfoils of owl, hawk, and NACA 0012 at two different chord lengths of 15 cm and 25 cm at different Reynolds numbers.

Aerodynamic efficiency (L/D) of six airfoils versus AOA for different Reynolds numbers ranging from Re = 44 × 10^3^ to Re = 160 × 10^3^ are presented in Fig. 5. As it is shown for chord 1, the highest L/D relates to Re = 44 × 10^3^ which is corresponds to higher lift at this condition, also the second one is Re = 160 × 10^3^ due to lowest drag. Interestingly, maximum angle of attack is for AOA = 6°. For NACA 0012 airfoil, AOA of maximum L/D are different by Reynolds number ranging from 4° to 8°. For Hawk airfoil it is understandable that the value of maximum L/D are very lower than two previous airfoils where the maximum L/D for hawk is 38.5 and for owl 68.9 And for NACA 0012 is 64.3. This lowering L/D is due to high separation of the hawk at this range of Reynolds number where is not corresponds to its flight regime. However, the owl is flight at this range of velocities specially for glide flight.

**Fig. 6:**
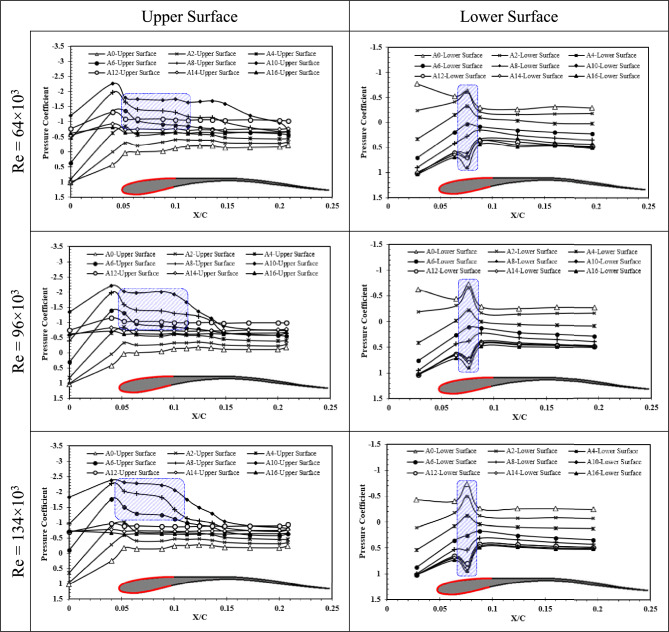
Pressure coefficient of upper surface and lower surface of owl airfoil at Reynolds numbers of Re = 64 × 10^3^, Re = 96 × 10^3^, and Re = 134 × 10^3^ different angles of attack.

As was previously explained, for understanding the physics of flow around three different airfoils, pressure measurements were also conducted on the airfoils of owl, NACA 0012, and hawk with Chord 2 (C = 25 cm). Figure 6 shows the Cp distribution on the upper and lower surfaces of owl airfoil at Re = 64 × 10^3^, Re = 96 × 10^3^, and Re = 134 × 10^3^ at angles of attack ranging from 0° to 16° by the step of 2°. The results of lower Reynolds number show that the –Cp at the upper surface is increased by increment of AOA before stall phenomenon occurring at AOA = 10°, which is in a good agreement with results obtained from force measurements. However, the constant pressure region (highlighted in the Fig. 6) at AOAs higher than AOA = 6°, reveals an onset of a laminar separation bubble on the upper surface of the owl airfoil at this Reynolds number. Also, for lower surface at this Reynolds number, an increment in the Cp at AOAs lower than 6° is visible which has a negative impact on the lift of the airfoil. However, for AOAs higher than 6°, this trend is reversed to a reduction in Cp. This interesting behavior can be happened due to the shift of LSB generated on the lower surface to upstream by increment of angle of attack. According to the results, the pressure distribution in the region of LSB for both upper and lower surface of airfoil for different Reynolds number remains constant.

**Fig. 7:**
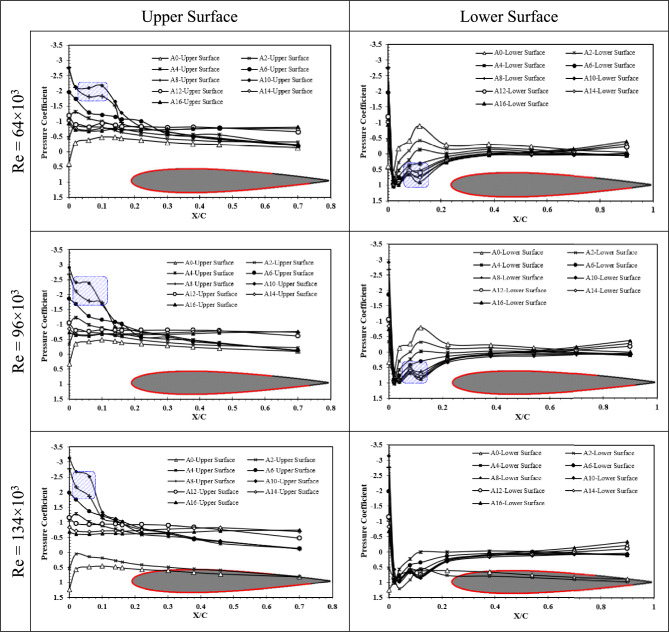
Pressure coefficient of upper surface and lower surface of NACA 0012 airfoil at Reynolds numbers of Re = 64 × 10^3^, Re = 96 × 10^3^, and Re = 134 × 10^3^ different angles of attack.

Furthermore, the Cp distribution on the upper and lower surfaces of NACA 0012 airfoil are presented in Fig. 7. Generally, same as owl airfoil, by increment of angle of attack, the upper surface Cp is reduced before it reaches its post-stall behavior of constant pressure distribution. From AOA = 8°, a constant region corresponding to LSB is observed that lasts until AOA = 10°, and its length is reduced by increment of Reynolds number. For lower surface of the NACA0012 airfoil, a high-pressure region is visible for AOA higher than 6° at Reynolds numbers of Re = 64 × 10^3^ and Re = 96 × 10^3^. This phenomenon could be onset of LSB on the pressure side of the airfoil which was also observed in the owl airfoil as well.

**Fig. 8:**
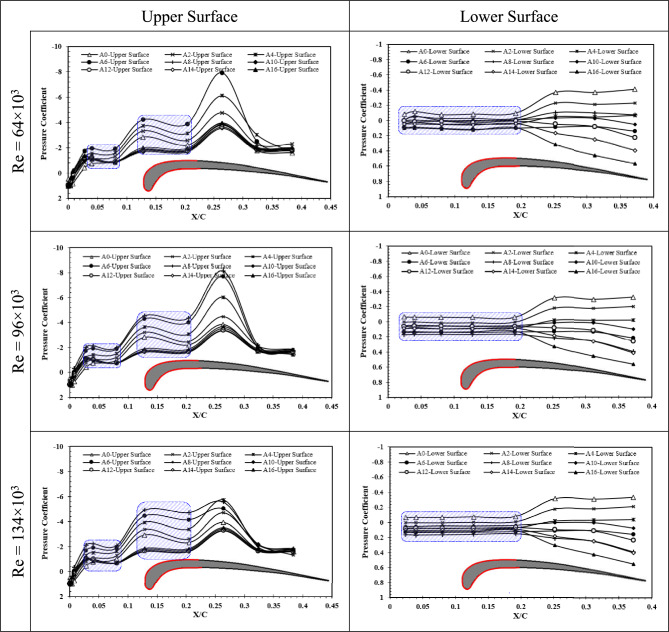
Pressure coefficient of upper surface and lower surface of Hawk airfoil at Reynolds numbers of Re = 64 × 10^3^, Re = 96 × 10^3^, and Re = 134 × 10^3^ different angles of attack.

The Cp distribution on the upper and lower surfaces of hawk airfoil are presented in Fig. 8. Obviously, the trend of Cp for this airfoil is not similar to other two airfoils and this is in response to the direct impact of high-cambered leading edge of this airfoil. Cp distribution of upper surface shows that the stall angle by increment of Re, delays from 6 to 8. Also, a LSB is visible at the leading edge before X/C = 0.1 and after that, again a constant region is visible between 0.1 and 0.2. This can imply that the LSB generated in upper surface of the airfoil consists of two different regions. Also, for lower surface of hawk airfoil, from leading edge up to X/C = 0.2 the flow is separated which is a long LSB generated on the lower surface, after that, the Cp is varied by AOA where the Cp has higher values by increment of AOA.

In conclusion, based on the results obtained from this section, the angle related to the stall and separation from the leading edge for both owl and NACA 0012 airfoils is higher than 10°, while for the hawk airfoil is 6°. This feature can have a great impact on noise generation in different phases of flight for hawk airfoil, where owl airfoil is very similar to a conventional typical symmetrical airfoil in this manner. Also, the LSB generation on the upper surface of owl starts from AOA = 6° to 10°, in NACA 0012 airfoil is generated from 8° to 10° degree, and for hawk airfoil, separation region started from the leading edge at AOA = 0°. Thus, from the point of view of the noise generated from aerodynamic sources, the hawk airfoil shows more susceptibility to aerodynamic noises compared to owl and NACA 0012 airfoil.

**Fig. 9:**
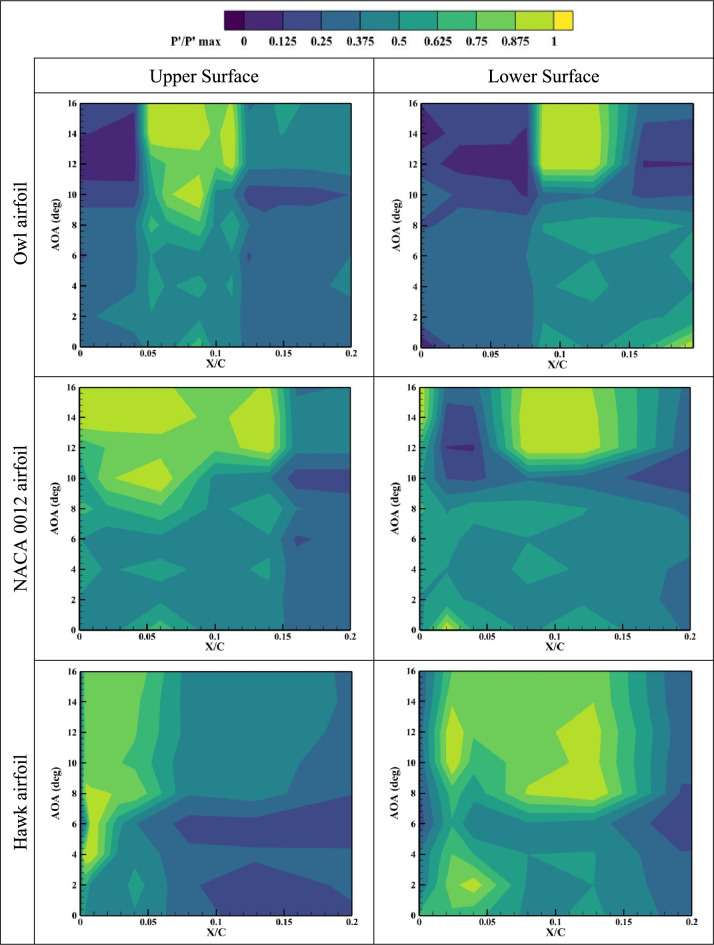
Normalized pressure fluctuations on the upper and lower surfaces of owl, NACA 0012, and hawk airfoils at Reynolds numbers of Re = 64 × 10^3^ in different X/C and AOA.

**Fig. 10:**
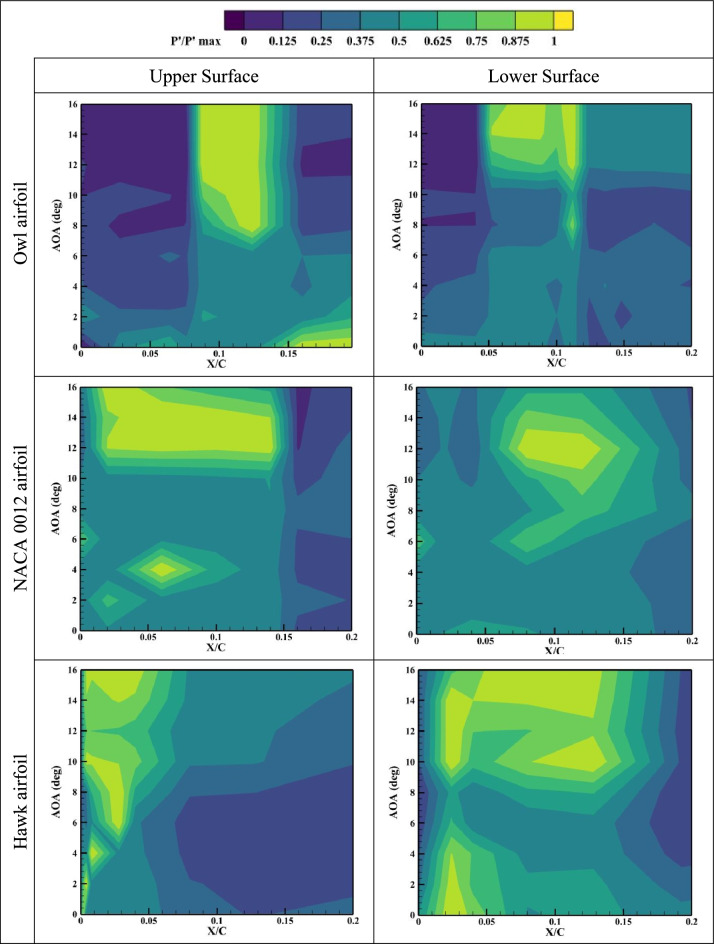
Normalized pressure fluctuations on the upper and lower surfaces of owl, NACA 0012, and hawk airfoils at Reynolds numbers of Re = 96 × 10^3^ in different X/C and AOA.

**Fig. 11:**
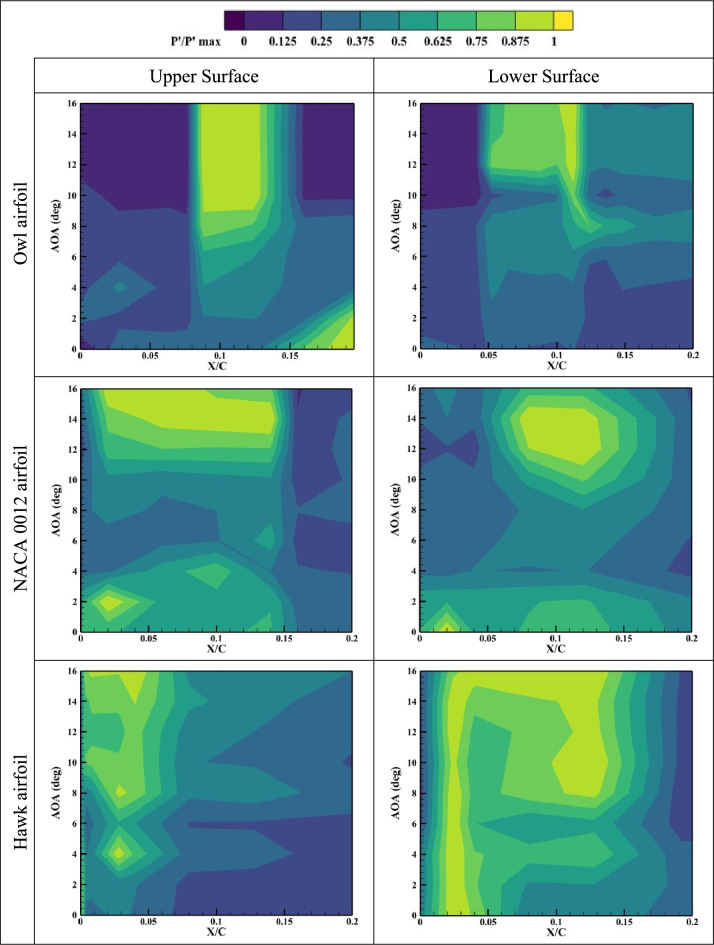
Normalized pressure fluctuations on the upper and lower surfaces of owl, NACA 0012, and hawk airfoils at Reynolds numbers of Re = 134 × 10^3^ in different X/C and AOA.

While in previous sections of this study, the mean aerodynamic properties for the specified airfoils were investigated in time-averaged sense, in this section, the RMS values of the suction and pressure side pressure fluctuations, Pʹ, is calculated for each pressure port for all angles of attack. Generally, laminar separation bubbles and separated shear flows contain regions of high recirculating flow, which can be detected through study^35^. Also, the region with the higher-pressure fluctuation values can be indicative of high aerodynamic noises. The results of normalized pressure fluctuations are presented in Fig. 9, Fig. 10, and Fig. 11 for three different airfoils of owl, NACA 0012, and hawk at different X/Cs and AOAs for Reynolds numbers of Re = 64 × 10^3^, Re = 96 × 10^3^, and Re = 134 × 10^3^. As is shown in Fig. 9, at lower Reynolds number, Re = 64 × 10^3^, the high-pressure fluctuations region of owl airfoil started from almost AOA = 6°. This result is aligned with the fact that an owl airfoil is identified as a silent flight airfoil for most of its flight regime specially in the cruise flight. However, for hawk airfoil this region started after AOA = 2°. In fact, the lower surface of the Hawk airfoil has a very large region for high fluctuations where could be a major source of aerodynamic noises. Also, the big high-pressure region of the lower surface of the hawk airfoil can be in response to the presence of a LSB as was also mentioned in previous results. Consequently, in-depth evaluation of pressure fluctuations reveals that the owl airfoil shows lower aerodynamic noise sources such as LSB, stall phenomena and separated shear layer on its lower surface. Also, the pressure fluctuations on the surface of the owl airfoil compared to NACA 0012 are presentative as a symmetrical and low phenomena airfoil, has lower high Pʹ region which is indicative of owl airfoil better performance. The results of higher Reynolds numbers show that by increment of Reynolds number, high pressure fluctuations in owl airfoil, is concentrated on a local region started from AOA = 6°, where other airfoils have more high-pressure fluctuations regions which are distributed in different locations and angles of attack.

**Fig. 12:**
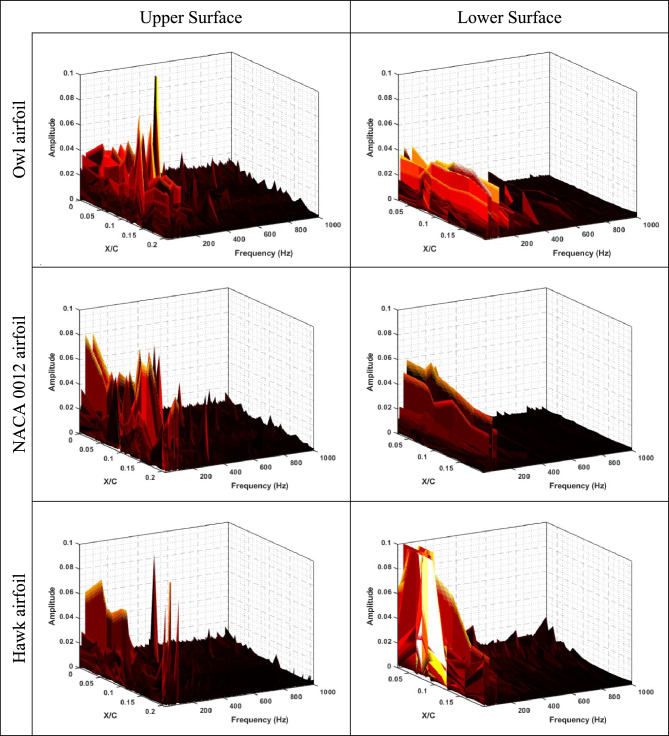
FFT of static pressure on the upper and lower surfaces of owl, NACA 0012, and hawk airfoils at Re = 64 × 10^3^ in different X/C and AOA = 8°.

**Fig. 13:**
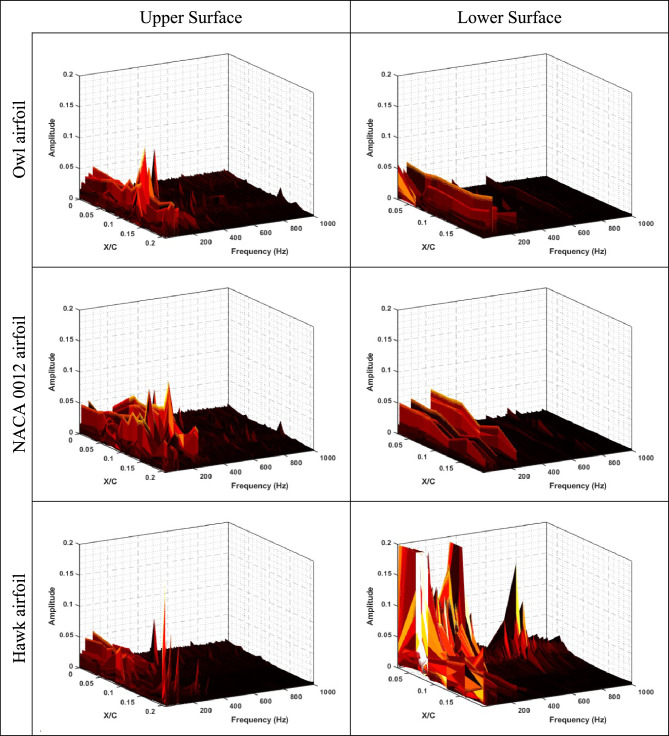
FFT of static pressure on the upper and lower surfaces of owl, NACA 0012, and hawk airfoils at Re = 96 × 10^3^ in different X/C and AOA = 8°.

**Fig. 14:**
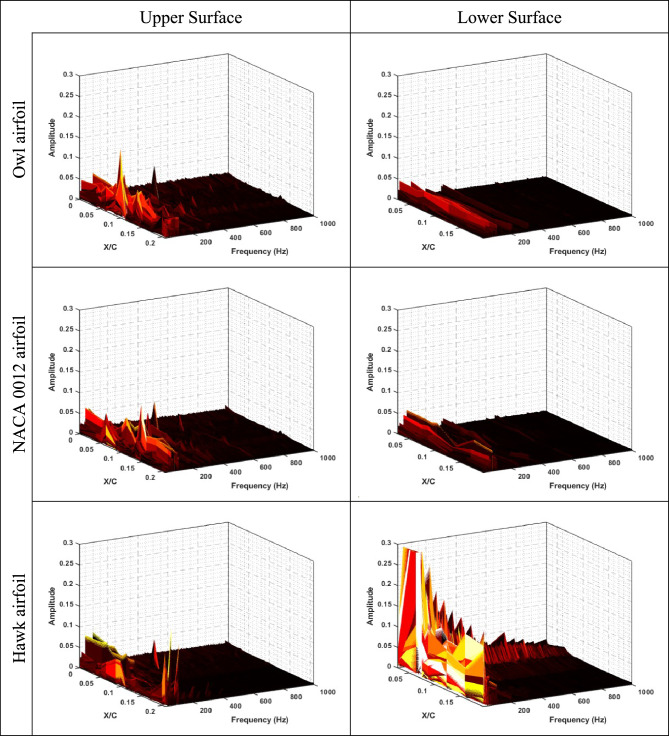
FFT of static pressure on the upper and lower surfaces of owl, NACA 0012, and hawk airfoils at Re = 134 × 10^3^ in different X/C and AOA = 8°.

To get further insight into physics of the flow around three different aforementioned airfoils (NACA 0012, Hawk airfoil, and Owl airfoil), frequency analysis was performed for static pressure fluctuations obtained on the upper and lower surfaces of airfoils. The results of frequency analysis are plotted in Fig. 12, Fig. 13, and Fig. 14 for Reynolds numbers of Re = 64 × 10^3^, Re = 96 × 10^3^, and Re = 134 × 10^3^, respectively for both lower and upper surface of the three airfoils.

As is shown in Fig. 12, it is concluded that main phenomena on the upper surface of owl airfoil have frequency near to 300 Hz which is a very high frequency for this test situations corresponding to free stream velocity of 4.8 m/s. from the point of view of starting vortex, the frequency of stating vortex shedding in this situation is f = V/C = 4.8/0.25 = 19.2 Hz, where the dominant frequency is larger from this frequency in the order of 10 times larger. Also, for the owl airfoil, the second dominant frequency is 250 Hz in the X/C = 0.075 for upper surface of the airfoil. This result is in agreement with the high-pressure fluctuation (Pʹ) region onset from the same X/C location in the contour of pressure fluctuations (Fig. 9). These two peak frequencies could be corresponding to the tonal noises for owl airfoil. Also, in this location, some small amplitude peak frequencies such as 20, 70, and 160 Hz are visible where the amplitude of all of those are lower than amplitude of peak frequency of 250 Hz. However, for lower surface of owl airfoil, a lower amplitude region on the entire surface is visible for frequency lower than 200 Hz and any peak value or phenomena is not visible. By comparing the FFT results of owl to the NACA 0012 and hawk airfoils, it is understandable that the amplitude of FFT for NACA 0012 and Hawk is higher than owl in average. Which corresponds to lower energy of the flow perturbations on the surface of the owl airfoil.

However, the results of frequency analysis for NACA 0012 airfoil show that this airfoil has aerodynamic noise sources in all locations of airfoil chord in contrast with the hawk airfoil which has four distinguished dominant peaks at the frequency of 20, 100, 120, and 180 Hz where all of them in the region of separation bubble for this airfoil from previous contours (P'). Also, the fluctuations with highest FFT amplitude which is could be the highest energy phenomena is for lower surface of hawk where has a high amplitude region in the leading edge and also, a broadband noise for all frequencies in the locations of lower than X/C < 0.1 and frequencies lower than 100 Hz.

To investigate the effect of free stream velocity or Reynolds number increment on the frequency domain analysis, Fig. 13, shows the results of FFT analysis for Re = 96 × 10^3^. As is illustrated in this figure, the location of peak values for owl airfoil remains constant by increasing Reynolds number; however, two peak frequencies of 100 and 180 Hz are also observed in this Reynolds number. However, it is expected that by increment of velocity, the Strouhal number (St) of phenomena being constant, by comparison of frequencies and Strouhal numbers of these frequencies, there is no meaningful relation visible compared to previous Reynolds number. It is shown that several parameters could affect the phenomena frequencies specially on the surface of the airfoil, at the low Reynolds number investigations. Also, for NACA 0012 airfoil and hawk airfoil, the explanation for lower Reynolds number is acceptable however for hawk airfoil, the phenomena with peak value of 20 Hz have higher amplitude in this Reynolds number, Also, lower surface of the hawk airfoil has higher amplitude from previous Reynolds number. It should be noted that the lower surface of hawk airfoil, contains very high amplitude at peak frequency of 770 Hz due to strong separation region on the lower surface. By increment of Reynolds number, it is visible at the Fig. 14, the location of frequency of 180 Hz, doesn’t change but the location of frequency of 100 Hz has been changed from X/C = 0.1 to X/C = 0.07. Other difference by increasing Re is that the lower surface of hawk airfoil, experiences much higher amplitude due to strong separation region.

## Conclusion

This study focuses on comparing the aerodynamic noise sources and aerodynamic performance of the owl airfoil with those of the NACA 0012 and hawk airfoils. The research involves several wind tunnel experiments utilizing pressure sensors and force balances to examine the aerodynamic noise sources and performance of the airfoils, considering two different chord lengths and varying free stream velocities. The investigation encompasses six airfoils (three airfoils with two different chord lengths) tested at different Reynolds numbers ranging from 44 × 10^3^ to 160 × 10^3^ to cover the glide flight range of owls across different species. The results obtained from the wind tunnel experiments are categorized and summarized as follows:

- The results of the aerodynamic performance comparison of the airfoils reveal that the owl airfoil exhibits a higher lift coefficient and L/D ratio, surpassing those of the NACA0012 and hawk airfoils by 6.7% and 44.1%, respectively. This characteristic enables owls to achieve efficient flights, particularly during the glide phase, at lower angles of attack where separations and fluctuations are minimized, resulting in lower aerodynamic noise levels.
- Furthermore, it is noteworthy that the stall angle for the owl airfoil is delayed with an increase in Reynolds numbers, ranging from 8° to 15°, while the NACA 0012 stalls at 10° to 11°, and the hawk airfoil exhibits stall angles varying from 6° to 12°. This observation further supports the notion that the owl airfoil may contribute to reduced aerodynamic noise levels by delaying the stall angle.
- From the results of pressure coefficient, it is concluded that the owl airfoil exhibits a generated LSB starting from AOA = 6° to 10°. In contrast, the hawk airfoil demonstrates the presence of an LSB from AOA = 0°. Consequently, from the perspective of noise generated from aerodynamic sources, the hawk airfoil displays greater susceptibility to aerodynamic noise compared to the owl and NACA 0012 airfoils.
- Through an investigation of the root mean square (RMS) of pressure fluctuations (P') on the airfoils, it is observed that the region of high-pressure fluctuations on the owl airfoil initiates at approximately AOA = 6°. Conversely, for the hawk airfoil, this region commences after AOA = 0–2°. Notably, the lower surface of the hawk airfoil exhibits a significantly large region of high fluctuations, potentially serving as a major source of aerodynamic noise. Consequently, a thorough evaluation of pressure fluctuations indicates that the owl airfoil exhibits fewer aerodynamic noise sources, such as laminar separation bubble, stall phenomena, and separated shear layer, on its lower surface.
- By examining the frequency domain of pressure fluctuations, it becomes evident that the amplitude of the FFT for the NACA 0012 and hawk airfoils is higher overall compared to that of the owl airfoil. This observation corresponds to lower energy of flow perturbations on the surface of the owl airfoil. Additionally, both the owl and hawk airfoils exhibit certain peak values in the FFT, corresponding to tonal noises, whereas the noises associated with the NACA 0012 airfoil are predominantly broadband.
- Investigation of the Reynolds number variation by changing the chord lengths and velocities shows two significant and surprising result. Firstly, it is noteworthy that for both chord lengths of the owl airfoil, the highest lift is obtained at a free stream velocity of 5.5 m/s (Re = 44 × 10^3^ for chord 1 and Re = 74 × 10^3^ for chord 2). This finding suggests that due to the high camber of the owl airfoil, the phenomenon at lower velocities does not follow the Reynolds number as a non-dimensional parameter. Secondly, the results of frequency domain analysis indicate that the location of peak values for the owl airfoil remains constant with increasing Reynolds number. However, it is expected that with an increase in velocity, the Strouhal number (St) of the phenomenon would remain constant. Upon comparison of frequencies and Strouhal numbers of these frequencies, no meaningful relationship is observed compared to previous Reynolds numbers. This suggests that several parameters could affect the frequencies of the phenomena, particularly on the surface of the airfoil, during low Reynolds number investigations.

In conclusion, the main characteristics of the owl airfoil, such as reduced noise and superior performance, make it suitable for applications like wind turbines, where quieter airfoils are needed, as well as for UAVs, due to its higher L/D ratio and ability to delay stall at low Reynolds numbers. Regarding the noise frequencies, it is important to highlight the relevance of this comparison in understanding how these frequencies in the air domain translate into the sound domain. Further investigation could examine how these frequencies relate to the auditory capabilities of the owl’s primary prey species, which could be explored in future studies.

## Data Availability

Raw data were generated by pressure sensors and force balance facilities at DANA aerodynamic laboratory. Derived data supporting the findings of this study are available from the corresponding author upon request.

## Abbreviations

AOA: Angle of attack
C: Airfoil chord (m)
CR: Nozzle contraction ratio
P′: Pressure fluctuation (P−P_avg_)
Re: Reynolds number (VC/ν)
TI: Turbulence intensity
St: Strouhal number (fC/V)
V: Free stream velocity (m/s)
